# Teamwork quality and health workers burnout nexus: a new insight from canonical correlation analysis

**DOI:** 10.1186/s12960-022-00734-z

**Published:** 2022-06-13

**Authors:** Wenxin Wang, Samuel Atingabili, Isaac Adjei Mensah, Hong Jiang, Hao Zhang, Akoto Yaw Omari-Sasu, Evelyn Agba Tackie

**Affiliations:** 1 Department of Public Administration, Law School, Shantou University, 243 Daxue Road, Shantou, Guangdong People’s Republic of China; 2 Institute of Local Government Development, Shan-Tou, 515063 People’s Republic of China; 3 School of Management, Jiangsu University, Zhenjiang, 212013 People’s Republic of China; 4 Institute of Applied Systems Analysis (IASA), School of Mathematics, Jiangsu University, Zhenjiang, 212013 People’s Republic of China; 5 Department of Population Health Sciences, Weill Cornell Medicine, New York, NY USA; 6 Department of Statistics and Actuarial Science, Kwame Nkrumah University of Science, Kumasi, Ghana

**Keywords:** Health workers, Burnout, Teamwork quality, Canonical correlation, China

## Abstract

**Background:**

Burnout is evidenced to have  adverse effect on the well-being of health workers. Although several risk factors of burnout have been found, only a hand full of studies have examined the role of teamwork quality. This study therefore sought to explore the relationship between the sub-dimensions of burnout and teamwork quality.

**Method:**

This is an empirical study involving health workers who have practising certificate from the National Health Commission of the People’s Republic of China. Relying on the study’s target population, a sample of 939 healthworkers complied to partake in the survey. Data were obtained from the administration of a well-structured electronic questionnaire containing the Maslach Burnout Inventory together with Healthy and Resilient Organization (HERO) scales correspondingly. The scales were then analysed using the canonical correlation approach (CCA).

**Results:**

The results unveiled a statistically significant correlation between teamwork quality and health worker burnout indicating that teamwork quality and burnout are canonically correlated. Further, examination on the relationship existing between the dimensions of teamwork quality and burnout unveiled that with the exception of personal accomplishment and teamwork dedication, teamwork quality sub-scales (teamwork vigour and teamwork absorption) were negatively related to emotional exhaustion and depersonalization as sub-scales of burnout, respectively.

**Conclusion:**

The study concluded that, surge in teamwork quality leads to reduced emotional exhaustion and reduced depersonalization while simultaneously increasing professional accomplishment. Therefore, this study presents a solid foundation for decreasing burnout syndrome in healthcare that can be implemented by successfully increasing levels of teamwork quality.

**Supplementary Information:**

The online version contains supplementary material available at 10.1186/s12960-022-00734-z.

## Background

The introduction and advancement of technology have made it possible for employees to work anytime and anywhere. Regardless of this technological advancement, teamwork quality is pivotal for providing quality and good healthcare delivery to patients at all levels of health facilities. Health workers barely perform their responsibilities or daily activities in separation. Their work is coordinated by members of the same organizations who belong to the same health unit or department and as well  share a common (mission, vision, job resources)—teamwork quality [1, 2] and experiences. Patient safety is strongly associated with the quality of teamwork when team members share care tasks, collectively work together, and communicate effectively amongst themselves [3]. The impression and thought that work is shared or supported by other colleagues should reduce burnout—emotional exhaustion and depersonalization [2]. Nonetheless, burnout is a symptom caused by work overload and stress that is becoming more common in human service occupations, particularly among professional health practitioners who work with patients [4–7]. Precisely, burnout is seen as an epidemic for healthcare workers in general [8]. Burnout has been identified in some occupational groups that involve hard work in public services, most commonly, those engaged in public health service delivery (doctors, nurses) [9]. Increasing job roles and workload resulting from frequent changes and innovation occurring within the primary health care practices affect health workers and their relationship with their work leading to burnout.

Emphatically, burnout does not only have a negative impact on health-care professionals' well-being, but it also leads to substandard patient treatment, as [7, 10, 11] has reported. Literature has made it clear that burnout has been regarded as an epidemic amid healthcare personnel in many economies globally. For instance, in the United States of America, Shanafelt [12] reported that 54.4% of health workers had at least one burnout symptom, whereas in New Zealand, the frequency of job burnout among doctors was as high as 50%. Further, a prevalence rate of 28% was recorded within the UK cohort group Oto-rhino-laryngologist [13]. In addition, a cross-sectional research of burnout among French medical professionals revealed 16.0% with a high emotional exhaustion level, 38.9% with a high personal accomplishment rate and 33.8% with a high depersonalization rate [14]. Similarly, a survey of Australian expert anaesthetics revealed that 20% experienced high emotional exhaustion, 20% of high depersonalization, and 36% experienced low personal accomplishment [15].^1^ However, in the case of China the problem is similarly prevalent among doctors, with prevalence rates ranging from 66.5 to 87.8% [16]. Chinese doctors, particularly those working at tertiary hospitals, overwork for long hours as a result of the country's large population and rising demand for health care [16, 17]. In China just 1.2 doctors provide healthcare services to per 1000 inhabitants, in comparison to 2.5 and 3.9, in the United States and Germany, respectively [7]. During the last three decades, the Chinese economy has grown significantly; yet, rising health-care demand, fuelled by an improved economy, has placed an undue stress on Chinese doctors [18, 19]. According to a recent study, the doctor-patient connection has seen a sudden decline, since one-third of Chinese doctors have had conflicts with patients, which as a result has led to significant rates of depressive symptoms and suicide attempts [20], all of which could be caused by burnout. Based on these discussed assertions,  it is  necessary to understand the state of burnout among Chinese doctors in order to develop measures for minimizing burnout and increasing health-care quality.

Although a considerable number of studies have been conducted pertaining to issues of burnout among health practitioners, researches in the case of Asia of which China is not exceptional is relatively limited compared to the West. Exemplarily, a cross-sectional study in Yemen reported that, 63.2 percent of respondent doctors recorded  high levels of emotional exhaustion, 19.4 percent reported high levels of depersonalization, whereas 33.0 percent reported low levels of personal accomplishment [21]. The burnout rate among Malaysian doctors was on the other hand was reported as 36.6 percent [22]. Further, a cross-sectional burnout survey among the Hong Kong public health workers showed that 31.4% of respondents met the high burnout criteria [23]. Based on a random two-levels linear regression approach, Li [17] conducted a study in Shandong province to explore the workplace characteristics that are linked to burnout among primary care professionals. Similarly, Gan [24] using a multiple linear regression technique examined burnout prevalence among general practitioners. Additionally, a cross-sectional survey conducted by Nie [25] in 7 hospitals within 3 provinces of China used a logistic regression analysis to critically examine the nexus between burnout and social support amid nurses. Moreover, by employing a multivariable regression analysis, Li [18] in Beijing–Tianjin–Hebei region of China investigated on the nexus between job satisfaction and burnout among anesthesiologists to determine the causal effects of the aforementioned variables. Lastly, a path analysis was used to investigate the prevalence of occupational burnout among Chinese health workers and as well as examine the effect of social support and role stress [26].

Irrespective of the limited number of studies conducted by previous researchers in China and its environs, teamwork quality sub-dimensions have not been adequately elucidated to the best of our knowledge with respect to their association with burnout. Moreover, because of diverse social, environmental, and cultural elements connected with teamwork quality, it is challenging to generalize findings from other literatures and as well make  assumptions in the Chinese context. Therefore, to fill in the gap the study in a multivariate framework analyses the nexus among two sets of variables (teamwork quality and burnout) and the interrelationships between their respective sub-scales using canonical correlation analysis (CCA) approach. Uniquely, the study is conceptualized from the revised job-demands resources (JD-R) model [27–29] which stipulates that every work has its own risks factors correlated with job-related stress. The JD-R model postulates that increased work demands contributes to increased stress levels and health problems. Hence, burnout is abstracted from job demand whereas teamwork is conceptualized as a job resource according to the revised job-demands resources model of burnout [27].

The paper’s remaining headings are structured as follows: Methods: introduces the data and methods used in this study; Results: presents results and Conclusion: discusses and concludes.

## Methods

Relying on significant contributions to humanity health needs and the entire country's economy, this extant study being an applied analysis, empirically focused on a survey from a hospital in Shenzhen, Guangdong province of China. Specifically, the study’s target population was 980 health workers who have practicing certificate from the National Health Commission of the People’s Republic of China. Of these, 939 (95.8%) working as health workers within the sampled hospital complied to partake in the survey. Specifically, the survey was conducted by electronic questionnaire, which contains interference items. Hence if an answer to an interference item in the questionnaire is wrong, the questionnaire will be determined to be invalid and will automatically be eliminated. A well-structured electronic questionnaire was used to analyse burnout using  the Maslach Burnout Inventory (MBI) scale^2^ [30] while the  teamwork quality was also analysed using  the Healthy and Resilient Organization (HERO) scale [31]. Comparatively, other measurement tools pertaining to teamwork quality do not take into consideration the health component when measuring teamwork quality. Precisely, the scale HERO illustrates how job characteristics influence psychological work adaptation elements, which ultimately affect the well-being and performance of employees. Further, the mentioned tool assesses healthy workplaces, taking into account employee health as well as organizational context elements (job demands) [32]. The HERO measurement scale for teamwork quality therefore deems appropriate to utilize in this extant study because it takes into consideration the individuals and the organization simultaneously as well as the health of team members which the study seeks to address. Prior to distributing the electronic questionnaires, the survey contained an invitation with an overview of the study and an informed consent form of the study questionnaire. Members who have practising certificate from the National Health Commission of the People’s Republic of China and  are health workers in the selected hospital received email invitations. The concepts of informed consent and confidentiality were applied to all participants in the survey.

### Construct measures

Health workers burnout and teamwork quality are not directly measured, but are derived from a set of individual measurement items or observed variables. Per the study’s conceptual framework, quality of teamwork among health workers was gauged using HERO scale [31]. Specifically, the mentioned construct contains three sub-scales (teamwork vigour, teamwork absorption and teamwork dedication) and have been assigned as explanatory variables in our study, whiles burnout which consist of three sub-scales (emotional exhaustion, depersonalisation and personal accomplishment), has been designated as a response variable. Generally, items measuring the sub-dimensions of  teamwork quality were scored using 5-point Likert scale where the response rate ranges from 1—strongly disagree to 5—strongly disagree. The items for emotional exhaustion and depersonalization sub-scales derived from the MBI scales (burnout sub-dimensions) were also based on a 5-point Likert scale. Again, the rate for responses pertaining to the items measuring personal accomplishment variably ranged from 0—never to 4—always, following the study of [33]. Detailed description of items measuring sub-dimensions of burnout and quality of teamwork constructs are reported in Additional file 1: Table S1.

Notably, following the study of [34], nine (9) items were used in measuring quality of teamwork, and the Cronbach’s alpha estimates for the three sub-scales (teamwork vigour, teamwork absorption and teamwork dedication) were 0.764, 0.722 and 0.746 correspondingly. Furthermore, in relation to the scale developed from the MBI scale, 22 measurement items were used to measure burnout among health workers. Employed items concerning the MBI inventory are classified into three dimensions (emotional exhaustion, depersonalization and personal accomplishment) with 0.802, 0.783 and 0.830 Cronbach’s alpha estimates correspondingly. All sub-scales of both constructs are characterized by Cronbach’s alpha greater than 0.70 indicating robust reliability for both scales for the study. Concerning the factor loadings, Chen [35] has reported that in factor analysis, the loadings of items measuring each construct should exceed 0.6. Thus, factor loadings pertaining to all the measurement items of the various constructs under study (teamwork quality and burnout) exceeds the threshold of 0.6 indicating that scales of measurement need to be maintained for further analysis. Also, convergent validities among the various constructs were measured using the average variance extracted (AVE). Notably, Hair [36], reported that, the estimated value of the AVE should not be less than 0.5. Relying on this assertion, there is sufficient evidence to clinch that there exist significant levels of convergence validity since all the estimates pertaining to the AVE test ranges from 0.610 to 0.711 hence greater than the aforesaid threshold of 0.5. Summarily, results pertaining to the factor loadings together with the Cronbach’s alpha and AVEs are outlined in Additional file 1: Table S2 from the Appendix.

### Analytical procedure

The analytical procedure’s initial phase featured an examination of the data distribution using the one-sample Kolmogorov–Smirnov test that unveiled that data obtained for the study do not  follow a normal distribution (*p* < 0.05). Following [37] study, we carried out the Herman single factor test by loading 31 items on a single factor. The aforementioned examination revealed that the total variance explained is 21.94%, (which is far less than 50%), indicating common method variance is not an issue in the data set. A descriptive analysis was then conducted with respect to study participants’ demographic characteristics, which can be inferred from Table 1. Further, a bivariate correlation analysis was conducted between the sub-dimensions of burnout and quality of teamwork scales distinctly. Finally, the multivariate relationships amid sub-scales of teamwork quality on burnout among health workers were scrutinized using the CCA technique. Precisely, the CCA was selected for this extant study, as it represents the highest level of generalized linear models and can as well be conceptualized as a method closely linked with the more widely used Pearson correlation coefficient. Compared to other methods, the CCA as a multivariate technique theoretically limits the probability of committing a Type I error anywhere within the study. More importantly, the CCA approach is employed to identify and measure associations among two-set of variables. Specifically, the CCA technique is more appropriate in the case where there are multiple intercorrelated outcome variables.

**Table 1:** Respondents demographic characteristics

Item	Scale	Frequency	Percentage
Gender	Male	369	39.30
	Female	569	60.7
Age	18–30	413	44.03
	31–45	380	40.51
	46–60	141	15.03
	60 +	4	0.43
Education	Undergraduate	505	53.80
	Master	234	24.90
	Ph.D	97	10.30
	Senior high/technical secondary/junior college	112	10.80
Marital status	Unmarried	317	33.80
	Married	603	64.30
	Divorced	18	1.90

### Canonical correlation analysis (CCA) framework

The CCA of Hotelling [38] is centred on the affiliation amid the linear combination of variables concerning the  sub-dimensions of teamwork denoted as U canonical variate and linear combinations of variables in the health worker burnout variable set represented as V canonical variate, such that the canonical affiliation among the two variables is canonically maximized. Emphatically, U and V canonical variables, which in our study stands for the relationship between teamwork quality sub-scales and sub-dimensions of health workers burnout, have been specified. The first combination can achieve the highest link of any combination regarding the original variables linearly. Other correlated pairs are then established under the constraint that they are not correlated with each previous pair following [39].

Theoretically, given a set of teamwork quality variables (${X}_{m\times p}$) and a set of health workers burnout variables (${Y}_{m\times q}$) the canonical variables for each set can be expressed as:

1 $$\mathrm{U}={\mathrm{m}}_{\mathrm{a}1}{\mathrm{X}}_{1}+{\mathrm{m}}_{\mathrm{a}2}{\mathrm{X}}_{2}+\dots +{\mathrm{m}}_{\mathrm{ap}}{\mathrm{X}}_{\mathrm{p}},$$

2 $$\mathrm{V}={\mathrm{n}}_{\mathrm{b}1}{\mathrm{Y}}_{1}+{\mathrm{n}}_{\mathrm{b}2}{\mathrm{Y}}_{2}+\dots +{\mathrm{n}}_{\mathrm{bq}}{\mathrm{Y}}_{\mathrm{q}},$$

where ${m}_{a1},\dots ,{m}_{ap}$ and ${n}_{b1},\dots ,{n}_{bq}$ represent the standardized canonical coefficients used in determining the redundant variables used in explaining the canonical variables. These standardized estimated parameters relatively exhibit the significance of the quality of teamwork set of variables in interpreting the variable set concerning  health worker burnout.

Specifically, the correlation coefficient ${(\delta }_{{U}_{i}{V}_{i}})$ used to investigate the inter-liaison between the two sets of variables between $U$ and $V$ is defined mathematically as:

3 $${\delta }_{{U}_{i}{V}_{i}}=\frac{Cov(U,V)}{\sqrt{\mathrm{Var}(U)Var(V)}};i=\mathrm{1,2},\dots ,p.$$

To examine the significance of the canonical correlation coefficient, the null conjuncture together with the alternative proposition are respectively expressed as:

$${H}_{o}:{\delta }_{1}={\delta }_{2}=\dots ={\delta }_{p}=0,$$

$${H}_{A}:{\delta }_{1}\ne {\delta }_{2}\ne \dots \ne {\delta }_{p}\ne 0.$$

Typically, the canonical correlation estimate cannot identify the proportion of variances explained by the predictor set in the criteria set. Nonetheless, computing the redundancy measure for each canonical correlation is an efficient method of solving such an issue.

### Summary of descriptive statistics

Summarily, Additional file 1: Table S3 from the appendix reports the descriptive statistics with respect to the mean, standard deviation, kurtosis and skewness pertaining to burnout (BO) and its sub-dimensions (emotional exhaustion (EE), personal accomplishment (PA) and depersonalization (DP)), together with teamwork quality (TW) and its sub-scales (teamwork vigour (TV), teamwork dedication (TD) teamwork absorption (TA)). Centering on the main constructs used, the explanatory variable of interest (TW) is evidenced to have the highest mean value of 4.287 with a dispersion parameter of 0.675 whereas the response variable (BO) is reported to have the least average value of 2.910 with a dispersion estimate of 0.393. Further, in the case of the sub-dimensions, EE is evidenced to have the utmost mean estimate of 3.119 with 0.779 dispersion coefficient compared to PA (M = 2.894, SD = 0.373) and DP (M = 2.664, SD = 0.657). Considering also the sub-scales of TW, TD featured the highest mean (4.377) with a standard deviation of 0.696 followed by TA (M = 4.283, SD = 0.712) with TV having the least estimated average value of 4.202 with a dispersion parameter estimate of 0.744. Specifically, in the case of skewness and kurtosis statistics, the general rule of thumb is that, for a series to follow a normal distribution the skewness together with the kurtosis test values are expected to be approximately 0 and 3 correspondingly [36]. Hence, PA and DP (as sub-dimensions of BO) as well as TW and all its sub-scales are witnessed to be negatively skewed whereas BO and its sub-scale EE are reported to be positively skewed. Interestingly, all the variables on the side of kurtosis are evidenced to be platykurtic in shape (meaning all the variables used have their respective kurtosis statistics to be less than the threshold of 3). With the affirmation the outlined outcomes, none of the kurtosis and skewness meets the conditions of normality for the study variables, thus we have strong evidence to conclude that all the series of observed variables do not follow the normal distribution. This thus supports the findings obtained the one-sample Kolmogorov–Smirnov test from the "Analytical procedure" section. With the data employed for the study not normally distributed, a non-parametric approach needs to be employed. This thus leads to the application of the canonical correlation approach. 

## Results

### Bivariate correlation analysis

The bivariate association summary indicating the linear relationships between burnout and teamwork quality sub-dimensions is presented in Table 2. Considering the correlation matrix, the highest correlation occurred between EE and DP (*r* = 0.349, *p* < 0.01) while the least correlation estimates were between PA and EE (*r* = 0.233, *p* < 0.01) in the case of burnout construct. For teamwork quality sub-dimensions, the correlation between TA and TD (*r* = 0.863, *p* < 0.01) showed the highest correlation while the smallest relationship is between TA and TSPV (*r* = 0.778, *p* < 0.01). Precisely, the correlation analysis performed can only reflect the linear relationship between the single variables pertaining to the constructs employed (health workers burnout and teamwork quality); hence, to reflect the overall correlation between the two-groups of variables, performing the CCA is of much importance.

**Table 2:** Bivariate correlation analysis

		EE	PA	DP	TV	TD	TA
EE	Pearson Correlation	1					
	Sig. (2-tailed)						
PA	Pearson Correlation	0.223^**^	1				
	Sig. (2-tailed)	0.000					
DP	Pearson Correlation	0.349^**^	0.277^**^	1			
	Sig. (2-tailed)	0.000	0.000				
TV	Pearson Correlation	0.011	0.007	0.004	1		
	Sig. (2-tailed)	0.729	0.819	0.898			
TD	Pearson Correlation	− 0.027	0.011	− 0.020	0.778^**^	1	
	Sig. (2-tailed)	0.406	0.734	0.531	0.000		
TA	Pearson Correlation	− 0.015	0.032	− 0.016	0.822^**^	0.863^**^	1
	Sig. (2-tailed)	0.643	0.329	0.627	0.000	0.000	

^**^ Correlation is significant at the 0.01 level (2-tailed). The bolded values indicate the highest and the smallest correlation coefficients between the respective traits. EE-emotional exhaustion, PA-personal accomplishment, DP-depersonalization, TV-teamwork vigour, TD-teamwork dedication, and TA-teamwork absorption. All the sub-dimensions were computed from the arithmetic mean scores of their respective measurement items

### Canonical correlation between health worker burnout and teamwork quality

Specifically, to examine the relationship between health workers burnout and quality of teamwork from a multivariate perspective, a multivariate test of significance is employed in the first place to check whether the full canonical model obtained from the analytical process is statistically significant or not. From Table 3, test statistics, including the Pillai’s, Hoteling, and Wilk’s lambda tests, all confirm that the full canonical function estimated to establish the multivariate relationship between sub-dimensions of burnout and teamwork quality is statistically significant.

**Table 3:** Multivariate test of significance, eigenvalue, and canonical correlations

Multivariate test of significance			
Test type	Value	Approximate *F*-value	Significant value
Pillais	0.326***	7.249	0.000
Hoteling	0.443***	8.939	0.000
Wilk’s lambda	0.315***	8.117	0.000

Eigenvalues and canonical correlations			
Function No	Eigenvalue	Canonical correlation	Squared Canonical correlation
1 -(${U}_{1}{V}_{1}$)	0.404	0.828	0.685
2 -(${U}_{2}{V}_{2}$)	0.023	0.149	0.022
3 -(${U}_{3}{V}_{3}$)	0.012	0.107	0.011

*** represents 1% level of significance

Additionally, the canonical correlation analysis leads to the extraction of three canonical functions considering eigenvalues and canonical correlation estimates. Specifically, the first canonical function is characterized by the highest eigenvalue of 0.404, a correlation estimate of 0.828, and a squared canonical correlation of 0.685, indicating a substantial shared variance of 68.5% between the first and second set of variables.^3^ We, therefore, agree on the first canonical function since canonical correlations, eigenvalues and the variance explained for the second and third canonical functions, are not substantial. The relationship amid the first pair of canonical variates (from the first canonical function) based on this outcome can be explained as portraying the maximum canonical correlation coefficient.

Relying on the first canonical function, the standardized canonical estimates for the canonical variables and the sub-dimension canonical loadings for the various canonical variates are further analysed. The results are, therefore, outlined in Table 4. Specifically, the magnitude concerning  the canonical weights (standardized canonical coefficients) shows the relative contributions of each sub-dimension in a specific set (being predictor or criterion) to the canonical variates. The canonical variables, which in our study are $U$ and $V$ exhibit the optimal linear combination among the predictor and criterion sets of variables. Thus, the ideal linear combinations for various sets of variables concerning Eqs. (1) and (2) can be estimated using the standardized canonical coefficients as:

**Table 4:** Standardized canonical coefficients and canonical loadings

Variable	Standardized Can. Coef	Structural coefficients (${{\varvec{r}}}_{{\varvec{s}}}$)	Canonical loadings
*Predictor set (teamwork sub-dimensions)*			
TV	− 0.719	− 0.593	− 0.615
TD	− 0.103	− 0.042	0.220
TA	− 0.628	− 0.463	− 0.391
*Criterion set (physician burnout sub-dimensions)*			
EE	0.817	0.959	0.912
PA	− 0.151	− 0.186	− 0.143
DP	0.316	0.502	− 0.084

Note: A variable with ${r}_{s}$ greater than $\left|0.45\right|$ is said to be significant and useful in the model. All values of ${r}_{s}$ greater than $\left|0.45\right|$ are therefore underlined

4 $${F}_{1}=\left\{\begin{array}{c}{U}_{1}=-0.719\mathrm{TV}+(-0.103)\mathrm{TD}+(-0.628)\mathrm{TP}\\ {V}_{1}=0.871\mathrm{EE}+\left(-0.151\right)\mathrm{PA}+0.316\mathrm{DP},\end{array}\right.$$

where ${F}_{1}$ represents the first canonical function,

As additionally outlined in Table 4, the loadings pertaining to teamwork quality unveiled that in forming ${U}_{1}$, teamwork vigour (TV) is the most dominant sub-dimension as compared to teamwork dedication (TD) and teamwork absorption (TA). Nonetheless, emotional exhaustion (EE) is also evidenced to be the most influential among the sub-scales of burnout when forming ${V}_{1}$. Estimation outcomes pertaining to the structure coefficients in Table 4 further measures the bivariate relationships between the dimensions of teamwork quality and that of burnout correspondingly. According to the report of Sherry and Henson [40], variables with structure coefficients greater than $\left|0.45\right|$ are classified to be highly useful and thus significant in the model. Thus structure coefficient values higher than aforementioned threshold has been underlined (see Table 4). We can therefore deduce that from the predictor set (teamwork quality dimensions), team work vigour and team absorption show high levels of usefulness whereas on the side of the criterion set, emotional exhaustion together with depersonalization are evidenced to be high degree of expediency. In the case of explaining the relationship amid the relevant dimensions of teamwork and burnout, it can be deduced that, both teamwork vigour and teamwork absorption (teamwork sub-scales) are negatively related with burnout sub-scales (emotional exhaustion and depersonalization) except personal accomplishment since the pairs of variables are characterized by structure coefficients with the different signs.^4^ Further, though teamwork dedication is evidence to be less useful in the quality of teamwork set (${r}_{s}$<$\left|0.45\right|$), it is also identified to be negatively related with emotional exhaustion and depersonalization with the exception of personal accomplishment.

Further, by looking at the cross-loadings of the various sub-dimensions with opposite canonical variables of the first canonical function, teamwork vigour, and emotional exhaustion contribute substantially to the canonical variables ${U}_{1}$ and ${V}_{1}$ correspondingly. Moreover, variance pertaining to the dependent and independent set of variables explained by the canonical variates is also examined. Outcomes from the variance analysis indicated that 68.6% of the total variance verified in the teamwork sub-dimension set of variables is accounted for by the first canonical variate (${U}_{1}$) with a redundancy estimate of 0.045, indicating 4.5% of the variance ratio is explained by the second canonical variable ${V}_{1}$. Also, the amount of variabilities in the sub-dimensions of burnout explained by the second canonical variable ${V}_{1}$, on the other hand, is determined as 20.8%, while the value of redundancy is obtained as 0.011 suggesting that 1.1% of the variance ratio is explained by the opposite canonical variable ${U}_{1}$. Summarily, results concerning both the cross-loadings and explained variance ratios are outlined in Table 5.

**Table 5:** Cross-loadings and total variance ratio explained

Cross-loadings of sub-dimensions with opposite canonical variables						
Canonical variable	Predictor set (teamwork sub-dimensions)			Criterion set (physician burnout sub-dimensions)		
	TV	TD	TA	EE	PA	DP
$\mathrm{U}$	–	–	–	0.790	− 0.006	− 0.017
$V$	− 0.471	0.215	− 0.109	–	–	–

Total variance ratio explained by canonical variables				
Canonical variable	Predictor set (teamwork sub-dimensions)		Criterion set (physician burnout sub-dimensions)	
	Variance explained	Redundancy	Variance explained	Redundancy
$\mathrm{U}$	0.686	–	–	0.011
$V$	–	0.045	0.208	–

## Discussion

Decreased workers’ morale and health professionals’ success levels negatively affect health institutions and can be linked to several factors. This may also involve administrative mistakes, but they are often connected to individuals’ tension and negative teachings and feelings. Job-related health service delivery has a structured effect on people’s cognition, and in many situations, these activities may improve short-term motivation while decreasing long-term motivation. The psychological issues that might arise from such interventions may lead to people leaving their jobs, according to [41]. In this context, from both a theoretical and realistic point of view, the relationship between burnout and work attitude and behaviour, which involves teamwork quality, is of great importance. Therefore, this recent study ensured to investigate the relationship between burnout and quality of teamwork in a multivariate framework via CCA method considering health worker staff in a selected  health facility in Shenzhen, Guangdong province of China.

Specifically, as [42] reported, the Cronbach alpha estimates range between 0.70 and 0.95. Corresponding test values of the Cronbach alpha obtained for the validity and reliability checks for the measurement constructs employed in the recent study were within the threshold. Therefore, the levels of the sub-dimensions for the various constructs (burnout and teamwork quality) were calculated based on the arithmetic mean from the scores of their respective measurement items.^5^ The relationships between the sub-dimensions concerning the respective study constructs using the bivariate correlation analysis were variably significant, thus in tandem with those reported in the researches [42, 43].

Although the sub-dimensions of teamwork quality are significant indicators for the emergence of health worker burnout, it is not easy to clarify the relationship between the sub-dimensions simultaneously using the bivariate correlation method [33]. Thus, as a substitute for construing the bivariate association, three canonical functions derived from the canonical connexion method were implemented to explain the interrelationship among the traits. These canonical functions were obtained because the number of canonical functions that need to be interpreted depends on construct with the minimum number of sub-dimensions. Thus, in the case of this study, each construct was measured using three sub-dimensions thus three canonical functions were obtained. Centring on the eigenvalues, canonical correlation, and squared canonical correlation estimates, only the first canonical function was deemed appropriate in explaining the association between quality of teamwork and health worker burnout in a multivariate framework.

Comparatively, outcomes from Table 4 in terms of structural coefficients unveiled that the dimensions of teamwork quality specifically teamwork vigour and teamwork absorption are negatively related with emotional exhaustion and depersonalization correspondingly. Specifically, the negative relationship evidenced between teamwork vigour and emotional exhaustion can be justified based on the fact that, health workers who express team work vigour in their line of duty may influence each other's moods and share various experiences because they are part of the same department or unit. That is, health workers who exhibit willingness to put forth efforts in working with a team, tends to persevere in the midst of adversity and in the long run experience less emotional exhaustion. These result findings are in line with study conducted by [34, 44]. Further, the negative affiliation between teamwork vigour and depersonalization could also mean that as health workers interact and share experience with members of their team, they feel a sense of belongingness, positive feeling and fulfilment. Health workers who are fond of isolating themselves from members of their unit and struggle to cope with work demands are able to overcome such challenges. The callous and pessimistic attitude towards an individual’s work and patients or other is reduced when team members express team vigour as evidenced in the study of [34, 45]. On the side of teamwork absorption, the negation associations evidenced with emotional exhaustions and depersonalization implies that, when health workers work as a team, members are able to remain completely focused and absorbed in their job. With time passing swiftly there is the difficulty in disconnecting themselves from the task at hand. Thus, when health workers exhibit team absorption they are able to generate proactive behaviours [32], experience less absent-mindedness [46], improve performance and higher patient retention. In a similar study conducted by García-Campayo, Puebla-Guedea [44] teamwork absorption provides emotional and psychological advantages to members by meeting a variety of individual requirements as well as the need for affiliation and integration in a group. Nonetheless, teamwork dedication though witnessed to be less relevant as a sub-scale of teamwork quality had a positive relationship with personal accomplishment but negatively affiliated to emotional exhaustion and depersonalization. Practically, the positive connexion between teamwork dedication and personal accomplishment may suggest that, team dedication may involve health workers showing full involvement in the work done by the team. Through this, health workers are able to identify with the task at hand as they feel a sense of increased competency and productivity. This according to [45, 47] can increase their self-esteem and reduce depression. On the other hand the negative rapport concerning the former (teamwork dedication) together with emotional exhaustion and depersonalization could thus indicate that health workers who work together and have comparable cognitive and behavourial tendencies, feel collective emotions, share collective efficacy, or share workload. They develop team members with shared beliefs and affective experiences who work hand in hand making individual members of the team experience less emotional exhaustion. This results is in tandem with the findings of [31] where the authors stated that when people and groups feel effective, they feel good in the short term (positive affect), and their involvement in their activity grows in the long run (exemplified by high effort, tenacity, dedication, and being completely immersed in the action). Health workers who experience depersonalization can manifest human behaviour towards patients or others when fully engaged in teamwork dedication.

Relying on the canonical correlation analysis outcomes, and the standardized canonical coefficients (canonical weights), the estimated effect of teamwork quality on health worker burnout is revealed. Notably, the outcome outlined in Table 5 infers that as the level of teamwork quality among health worker staff increases, emotional exhaustion and depersonalization decreases while inflating professional accomplishment. Practically this implies that, teams have a common purpose which is oriented towards achieving a particular objective. Hence, once there exist team spirit among health workers working in the same department, they are able to provide psychological and emotional benefits to individual members of their team which reduces their level of emotional exhaustion and depersonalization. As a result, health workers appreciate the necessity of teamwork quality to intensify morale and service provision leading to increase their level of personal accomplishment. This finding supports the study of [33] and [48]. Summarily, outcomes from the canonical correlation technique; lend to support the principle that teamwork vigour is the primary determinant of burnout among health workers.

**Fig. 1:**
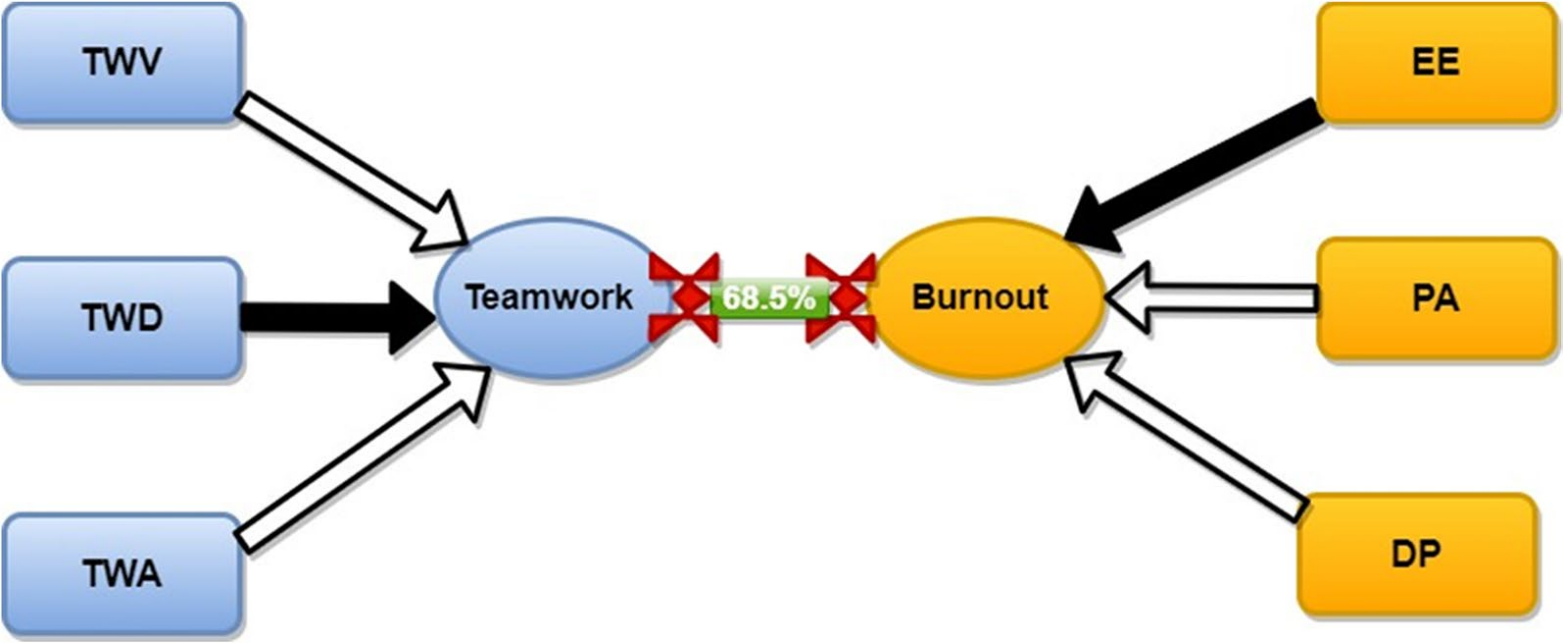
Pictorial illustration of the Health Resilient Organization (HERO) canonical loadings scales on the first predictor canonical variates and the canonical loadings of the Maslach Burnout Inventory scales on the first criterion variate and the association between teamwork and physician burnout. White arrows represent negative correlations, and black arrows denote corresponding positive correlations

Emphatically, variables (sub-dimensions) with larger canonical weights are regarded to contribute substantially to the multivariate relationship between the sub-dimensions of teamwork quality and health worker burnout (refer to Table 5). This reinforces the notion that teamwork vigour should be recognized as the paramount factor in explaining the emergence of burnout among health workers relative to the other sub-dimensions of teamwork quality. Our evidence is therefore in tandem with those reported by [33] and [49]. The canonical correlation coefficient between the two sets of variables (teamwork and health worker burnout) was therefore found to be 68.5%. This therefore gives a clear ability of this study to recognize the multivariate relationship between teamwork and health worker burnout. Figure 1 therefore displays the canonical loadings of the Health Resilient Organization (HERO) scales on the first predictor canonical variates, and the canonical loadings of the Maslach Burnout Inventory [30] scales on the first criterion variate and as well shows the association between teamwork quality and health worker burnout.

## Conclusion

Health service professionals of which health workers are not exceptional provide patients with a high degree of satisfaction while their work is challenging. Psychological issues such as burnout condition may arise from uncontrollable levels of stress. Thus, it should be noted that burnout among workers in the health service can adversely affect the quality of healthcare delivery and healthcare professionals’ quality. While it is taught that teamwork quality is mainly an idea that decreases the probability of people leaving an organization and promotes communication between employees, it is also recognized as an idea that affects burnout. Thus, relying on this assertion, the current study examined the relationship between the sub-dimensions of teamwork quality and burnout among health workers in a multivariate framework using CCA as the main statistical technique.

Major outcomes from the CCA approach unveiled that quality of teamwork, which is a predictor, leads to reduce emotional exhaustion together with depersonalization while at the same time increasing professional accomplishment. Further, teamwork vigour as a sub-dimension of teamwork construct was a primary determinant of burnout among health workers. Examination concerning the relationship between the different dimensions of teamwork quality and burnout additionally unveiled that, both teamwork vigour and teamwork absorption have a negative liaison with emotional exhaustion and depersonalization correspondingly whereas teamwork quality though witnessed to be insignificant in terms of its structural coefficient was similarly evidenced to be negatively related to emotional exhaustion and depersonalization. Finally, the canonical correlation coefficient of 0.685 indicated a significant canonical correlation between the predictor set of variables (teamwork quality) and the criterion set of variables (health worker burnout).

Relying on the outlined significant findings from the CCA approach, this present survey presents a solid foundation for decreasing burnout syndrome in healthcare that can be implemented by successfully increasing quality teamwork levels. Promoting human interaction to reverse or avoid emotional exhaustion will build health workers’ opportunities to excel and prevent burnout. To make this possible, increasing the number of available resources and increasing opportunities for social activities. The introduction of regular team meetings at which suggestions and criticisms are welcomed and creating a permanent mechanism to aid future problem-solving needs to be implemented in the various health organizations in the province.

## Supplementary Information

**Additional file 1.** Summary description of constructs and measurement items.

## Data Availability

The datasets generated and/or analysed during the recent study are not publicly available due to ethical issues related to respondents’ confidentiality.

## Abbreviations

CCA: Canonical correlation analysis
CMDA: Chinese Medical Doctors Association
MBI: Maslach Burnout Inventory
HERO: Healthy and Resilient Organization
EE: Emotional exhaustion
DP: Depersonalisation
PA: Personal accomplishment
TA: Teamwork absorption
TD: Teamwork dedication
TV: Teamwork vigour
KMO: Kaiser–Meyer–Olklin
PCA: Principal component analysis

## Footnotes

1 According to Maslach et al. [30], emotional exhaustion, depersonalization, and personal accomplishment are classified as sub-dimensions of burnout constructs.

2 The MBI Chinese version was employed.

3 The explained variance is represented in unique variance by the squared canonical correlation for each canonical function.

4 Specifially, the structure coefficient of both teamwork vigour and teamwork absorption are negative, whereas that of emotional exhaustion and depersonalization are positive. This therefore justifies the negative relationship between the pairs of variables.

5 Items with weightier loading from the factor analysis through the PCA method were used and the outcomes are presented in Additional file 1: Table S2.
