# Supplementary material for: Teamwork quality and health workers burnout nexus: a new insight from canonical correlation analysis
Source: Hum Resour Health. 2022 Jun 13;20:52. doi: 10.1186/s12960-022-00734-z (PMC9190088; doi:10.1186/s12960-022-00734-z)
Supplement: Supplementary file 1 — Additional file 1. Summary description of constructs and measurement items. [file 12960_2022_734_MOESM1_ESM.docx]

**APPENDIX**

**Table A1. Constructs and measurement items summary**

| Construct | Indicator | Measurement items | Scale |
| --- | --- | --- | --- |
| **Physician burnout** |  |  |  |
| Emotional exhaustion | EE1 | The hospital work made me tired | Ordinal [1-5] |
|  | EE2 | I get sick and tired of questions from patients and families | Ordinal [1-5] |
|  | EE3 | I worry that work would affect my mood | Ordinal [1-5] |
|  | EE4 | I often feel exhausted | Ordinal [1-5] |
|  | EE5 | At the end of the day, I feel very tired | Ordinal [1-5] |
| Professional accomplishment | PA1 | I can effectively solve patients' problems | Ordinal [1-5] |
|  | PA2 | My patients often complain about me | Ordinal [1-5] |
|  | PA3 | I can effectively influence others through work | Ordinal [1-5] |
|  | PA4 | I work with coping attitude | Ordinal [1-5] |
|  | PA5 | I have the ability to create a relaxed work environment. | Ordinal [1-5] |
|  | PA6 | I get really excited when I solve patients' problems | Ordinal [1-5] |
|  | PA7 | I often make small mistakes in my work | Ordinal [1-5] |
|  | PA8 | I would like to study or work by myself | Ordinal [1-5] |
|  | PA9 | I worry that patients are not happy with the way I handle problems. | Ordinal [1-5] |
| Depersonalization | DP1 | I often blame my patients | Ordinal [1-5] |
|  | DP2 | I have done a lot of meaningful works | Ordinal [1-5] |
|  | DP3 | I am not interested in scientific research | Ordinal [1-5] |
|  | DP4 | I felt nervous and afraid after a medical mistake | Ordinal [1-5] |
|  | DP5 | I always treat patients with caution | Ordinal [1-5] |
| **Teamwork** |  |  |  |
| Teamwork vigor | TV1 | During the task, my team feels full of energy | Ordinal [1-5] |
|  | TV2 | Willingness to do extra work without pay | Ordinal [1-5] |
|  | TV3 | Encouraging members to promote active participation | Ordinal [1-5] |
| Teamwork dedication | TD1 | My team is enthusiastic about the task | Ordinal [1-5] |
|  | TD2 | Information sharing and problem solving | Ordinal [1-5] |
|  | TD3 | Cooperating with superiors to complete work | Ordinal [1-5] |
| Teamwork absorption | TA1 | When my team is working, we forget everything else around us | Ordinal [1-5] |
|  | TA2 | Job changes acceptance | Ordinal [1-5] |
|  | TA3 | Maintenance of a good cooperative relationship | Ordinal [1-5] |

**Table A2**: Factor loadings and reliability analysis

| **Construct** | **Indicator** | **Measurement Items** | **Factor loadings** | **Cronbach's alpha** | **Average variance extracted (AVE)** |
| --- | --- | --- | --- | --- | --- |
| **Burnout** |  |  |  |  |  |
| Emotional exhaustion | EE |  |  | 0.802 | 0.711 |
|  |  | The hospital work made me tired | 0.808 |  |  |
|  |  | I get sick and tired of questions from patients and families | 0.618 |  |  |
|  |  | I worry that work would affect my mood | 0.695 |  |  |
|  |  | I often feel exhausted | 0.816 |  |  |
|  |  | At the end of the day, l feel very tired | 0.791 |  |  |
| Professional accomplishment | PA |  |  | 0.783 | 0.680 |
|  |  | My patients often complain about me | 0.624 |  |  |
|  |  | I can effectively influence others through work | 0.679 |  |  |
|  |  | I work with a coping attitude | 0.619 |  |  |
|  |  | I have the ability to create a relaxed work environment | 0.639 |  |  |
|  |  | I get really excited when l solve a patients' problem | 0.682 |  |  |
|  |  | I often make small mistakes in my work | 0.681 |  |  |
|  |  | I would like to study or work by myself | 0.632 |  |  |
|  |  | I worry that patients aren't happy with the way I handle problems | 0.633 |  |  |
| Depersonalization | DP |  |  | 0.830 | 0.693 |
|  |  | I often blame my patients | 0.620 |  |  |
|  |  | I'm not interested in scientific research | 0.663 |  |  |
|  |  | I felt nervous, afraid after making a medical mistake | 0.848 |  |  |
|  |  | I always treat patients with caution | 0.884 |  |  |
| **Teamwork** |  |  |  |  |  |
| Teamwork vigor | TV |  |  | 0.764 | 0.716 |
|  |  | Willingness to do extra work without pay | 0.668 |  |  |
|  |  | Encouraging members to promote active participation | 0.711 |  |  |
|  |  | During the task, my team feels full of energy | 0.632 |  |  |
| Teamwork dedication | TD |  |  | 0.722 | 0.610 |
|  |  | Information sharing and problem-solving | 0.827 |  |  |
|  |  | Cooperating with superiors to complete work | 0.837 |  |  |
|  |  | My team is enthusiastic about the task | 0.819 |  |  |
| Teamwork absorption | TA |  |  | 0.746 | 0.601 |
|  |  | Job changes acceptance | 0.795 |  |  |
|  |  | Maintenance of a good cooperative relationship | 0.816 |  |  |
|  |  | When my team is working, we forget everything else around us | 0.749 |  |  |

**Table A3:** Summary of descriptive statistics

| Variables |  | Mean  Statistic | Std. Deviation  Statistic | Skewness  Statistic | Kurtosis  Statistic |
| --- | --- | --- | --- | --- | --- |
| **BO** |  | **2.910** | **0.393** | **0.111** | **0.929** |
| EE |  | 3.119 | 0.779 | 0.190 | -0.252 |
| PA |  | 2.894 | 0.373 | -0.162 | 1.376 |
| DP |  | 2.664 | 0.657 | -0.095 | -0.214 |
| **TW** |  | **4.287** | **0.675** | **-1.138** | **1.317** |
| TV |  | 4.202 | 0.744 | -1.034 | 1.242 |
| TD |  | 4.377 | 0.696 | -1.195 | 1.206 |
| TA |  | 4.283 | 0.712 | -1.008 | 0.723 |

Note: POS= perceived organizational support, BO=burnout, EE=emotional exhaustion, PA=personal accomplishment, DP=depersonalization, TW=teamwork, TV=teamwork vigor, TD= teamwork dedication, TA=teamwork absorption. Further, EE, PA and DP are subscales of BO whereas TV, TD and TA are sub-scales of TW. Variables including BO and its sub-scales (EE, PA &DP) together with TW and its sub-scales (TV, TA &TD) are transformed using the arithmetic means from the scores of their respective measurement items.
